# Progranulin’s Protective Mechanisms and Therapeutic Potential in Cardiovascular Disease

**DOI:** 10.3390/cells14110762

**Published:** 2025-05-22

**Authors:** Gan Qiao, Yongxiang Lu, Jianping Wu, Chunyang Ren, Roudian Lin, Chunxiang Zhang

**Affiliations:** 1 Department of Pharmacology, School of Pharmacy, Southwest Medical University, Luzhou 646000, China; dqz377977905@swmu.edu.cn (G.Q.); luyx0807@163.com (Y.L.); 17865681795@163.com (J.W.); swmurcy@163.com (C.R.); 13711690551@163.com (R.L.); 2 Nucleic Acid Medicine of Luzhou Key Laboratory, Central Nervous System Drug Key Laboratory of Sichuan Province, Southwest Medical University, Luzhou 646000, China; 3 Key Laboratory of Medical Electrophysiology, Ministry of Education and Medical Electrophysiological Key Laboratory of Sichuan Province, (Collaborative Innovation Center for Prevention of Cardiovascular Diseases), Institute of Cardiovascular Research, Southwest Medical University, Luzhou 646000, China

**Keywords:** progranulin (PGRN), cardiovascular disease (CVD), protective mechanisms, signaling pathways, therapeutic target

## Abstract

Cardiovascular disease (CVD) remains a leading cause of morbidity and mortality globally, prompting the investigation of novel therapeutic targets. Progranulin (PGRN), a glycoprotein initially associated with neurodegenerative disorders, has emerged as a critical protective agent in cardiovascular health. Recent studies indicate that PGRN exerts its protective effects through various mechanisms, including the modulation of inflammatory pathways, enhancement of mitochondrial function, and promotion of vascular integrity. By engaging with key signaling pathways, such as PI3K/Akt, NF-κB and Wnt/β-catenin, PGRN mitigates oxidative stress and fosters an environment conducive to cardiac repair following ischemic injury. Furthermore, PGRN’s role in lipid metabolism and vascular smooth muscle cell behavior highlights its complexity in influencing atherogenesis and vascular homeostasis. This review synthesizes current knowledge regarding PGRN’s protective mechanisms in CVD, emphasizing its potential as a therapeutic target and paving the way for innovative approaches to prevent and treat cardiovascular diseases, ultimately improving patient outcomes in this critical area of public health.

## 1. Introduction

Cardiovascular disease (CVD) remains a predominant cause of morbidity and mortality globally, necessitating urgent attention to the identification of novel therapeutic targets and strategies to alleviate its burden [1]. Among the emerging candidates, progranulin (PGRN), a glycoprotein initially recognized for its role in neurodegenerative disorders, has garnered significant interest due to its multifaceted biological functions within the cardiovascular system [2,3]. Recent studies have illuminated PGRN’s critical involvement in the regulation of inflammation, cellular repair, and metabolic processes, positioning it as a potential protective agent in cardiovascular health [4,5]. The complexity of PGRN’s actions, particularly its interactions with key signaling pathways, such as PI3K/Akt and NF-κB, suggests that it plays a vital role in mitigating oxidative stress and fostering an environment conducive to cardiac repair following ischemic injury.

Emerging evidence indicates that PGRN exerts its protective effects through various mechanisms, including the modulation of inflammatory pathways, enhancement of mitochondrial function, and promotion of vascular integrity [6,7]. Its influence extends to lipid metabolism and vascular smooth muscle cell behavior, underscoring the intricate interplay between PGRN and cardiovascular homeostasis. Notably, PGRN levels have been identified as significant predictors of risk in patients experiencing acute myocardial infarction, highlighting its potential as both a biomarker for cardiovascular disease severity and a therapeutic target for intervention [3].

This review aims to synthesize current knowledge regarding the protective mechanisms of PGRN in CVD, emphasizing its relevance in the various cardiac pathologies, including myocardial infarction, sepsis-induced cardiomyopathy, and diabetes-induced cardiomyopathy. By elucidating the diverse roles of PGRN and its implications for cardiovascular health, we seek to pave the way for innovative therapeutic approaches that could enhance patient outcomes in this critical area of public health. The exploration of PGRN’s protective mechanisms represents a promising frontier in the ongoing quest to combat cardiovascular disease and improve overall cardiovascular health.

## 2. The Structure and Function of Progranulin

**Figure 1:**
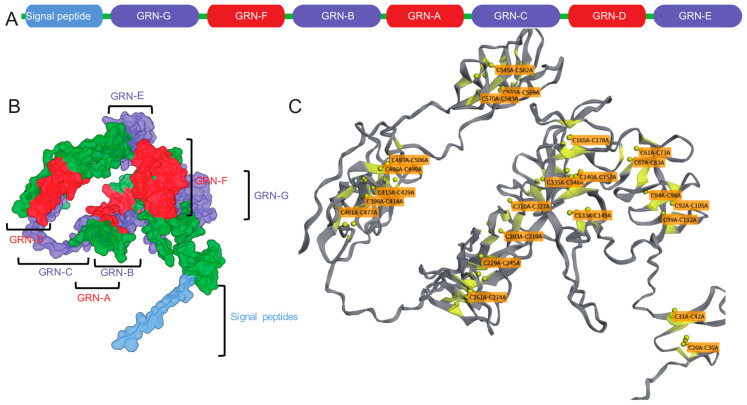
The complexity of Progranulin (PGRN). (**A**) The linear arrangement of PGRN’s components, starting with a signal peptide followed by seven and a half granulin domains (GRN-G, F, B, A, C, D, E) in the order P-G-F-B-A-C-D-E. (**B**) A three-dimensional model of PGRN, with each domain color-coded to match the linear sequence, demonstrating the protein’s intricate folding. Labels indicate the location of specific granulin domains and the signal peptide within the 3D structure. (**C**) A structural representation highlighting the crucial disulfide bonds (represented by yellow spheres) that maintain each granulin domain’s structural integrity. The labels indicate the cysteine residues involved in these disulfide bridges, which are essential for forming the characteristic β-sheet structure surrounding an axial core in each granulin peptide. This arrangement was predicted using the Biotite Python package.

The human GRN gene, located on chromosome 17q21.31, encompasses a tightly packed coding region 8 kilobases (kb) in length [8]. This gene encodes PGRN, a glycoprotein composed of 7.5 granulin units arranged in the sequence P-G-F-B-A-C-D-E [9], as shown in Figure 1A. The 3D structure of PGRN is shown in Figure 1B. The structural organization of these granulin motifs is characterized by a high cysteine content, which contributes to the stability and functionality of the protein [10]. Full-length human PGRN is a 593-amino-acid protein (~68.5 kDa theoretical) that, upon N-linked glycosylation at four confirmed and one putative sites, is secreted as an ~88 kDa glycoprotein [11]. Notably, PGRN can be cleaved by elastase into smaller peptides known as granulins, which possess a molecular weight of 6–8 kDa and are defined by a structure comprising four β-sheets surrounding an axial rod stabilized by 5–6 disulfide bonds [12]. The disulfide bond arrangement in PGRN was predicted using the Biotite Python package (version 1.3) and is shown in Figure 1C (the visualization code is publicly available on GitHub—https://github.com/GanQiao1990/disulfide_bridges_analysis (accessed on 1 May 2025)). This extensive disulfide cross-linking confers remarkable stability to the granulins in the oxidizing extracellular milieu, which supports their functional role in cardiovascular tissue remodeling and inflammatory regulation.

The diverse biological functions of PGRN are intricately linked to its structural characteristics, particularly the functional domains within its granulin motifs that facilitate interactions with various receptors [13]. The C-terminal granulin E domain is essential for the stable binding of PGRN to the β-propeller tunnel of sortilin (SORT1), a process critical for its transport to lysosomes for degradation in neuronal cells [14]. Additionally, PGRN’s interaction with intermediate molecular chaperones, such as endoplasmic reticulum protein 57 (ERp57), aids in the recruitment of heat shock protein 70 (Hsp70) to glucocerebrosidase (GCase), thereby playing a role in the pathophysiology of Gaucher’s disease [15,16]. Furthermore, PGRN interacts with ras-related protein 2 Rab2 (Rab2), a pivotal molecule in autophagosome-lysosome fusion, through a 15 kDa fragment containing the granulin E domain, thereby regulating autophagic flux [16]. PGRN has also been identified as a novel ligand of the tumor necrosis factor (TNF) family, forming a hetero-hexameric complex with three tumor necrosis factor receptor (TNFR) binding domains [17]. This interaction is crucial for immune cell activation within inflammatory microenvironments [18,19]. The three binding domains of PGRN give rise to atsttrin, a promising derivative for drug development, which maintains its affinity for TNFR despite structural sequence alterations [20].

Recent studies have highlighted the implications of the c.893G > A mutation in the GRN gene, resulting in a p.R298H substitution [21]. This mutation has been associated with a spectrum of clinical phenotypes, including cognitive decline, atrophy, and muscle weakness, underscoring the pathogenicity of GRN-related disorders. The functional domains of PGRN are thus subject to transformation, enabling precise interactions with various proteins and facilitating the regulation of cellular biological behaviors under specific conditions.

## 3. Progranulin and Cardiovascular Disease

### 3.1. Mechanisms of Progranulin’s Impact on Cardiac Pathologies

#### 3.1.1. Myocardial Infarction and Ischemia-Reperfusion Injury

Myocardial infarction (MI) is a critical cardiovascular event characterized by the interruption of blood flow to the myocardium, resulting in ischemia and subsequent necrosis of heart tissue [22,23,24]. This condition is a leading cause of sudden cardiac death and heart failure among patients with cardiovascular disease. Therapeutic interventions to restore blood flow, such as percutaneous coronary intervention (PCI) and thrombolysis, are widely used but can paradoxically induce ischemia–reperfusion (I/R) injury, wherein restoration of blood supply exacerbates myocardial damage and increases the risk of complications, including cardiac rupture and heart failure [25].

**The studies** have identified serum levels of PGRN as a significant predictor of risk in patients experiencing acute MI. For instance, cohort research demonstrated that serum PGRN concentration is positively correlated with the severity of coronary artery disease (r = 0.362, *p* = 0.0001) and serves as a risk predictor in acute MI patients [26].

A further study elucidated the role of PGRN in the pathophysiology of MI and I/R injury. In animal models, PGRN deficiency exacerbates post-MI remodeling, leading to increased mortality, heightened left ventricular fibrosis, and a higher incidence of severe arrhythmias. PGRN is expressed in cardiac tissue and infiltrating macrophages, particularly at the border zones of infarcted myocardium, where its absence leads to increased macrophage infiltration, fibrosis, and adverse post-MI outcomes [3]. Notably, PGRN expression increases progressively in the heart within 1, 3, and 7 days after MI in these models. Neutrophils secreting PGRN in the infarcted myocardium serve to limit further neutrophil migration, dampening the inflammatory response and reducing tissue damage, which ultimately leads to reduced infarct size and improved cardiac function after injury [27]. Additionally, administration of PGRN in rat models significantly reduces episodes of ventricular tachycardia and fibrillation following acute myocardial ischemia–reperfusion injury, indicating a protective role in arrhythmogenesis [28]. Furthermore, experimental models of I/R injury, including middle cerebral artery occlusion in mice, show that reduced PGRN expression exacerbates tissue damage, while treatment with recombinant PGRN (r-PGRN; Recombinant Mouse Progranulin) significantly decreases infarct size and brain swelling, lowering mortality rates [29].

Experimental studies further reveal that PGRN regulates macrophage activation and infiltration by modulating the expression of markers, such as CD206 and MerTK, thereby promoting reparative macrophage infiltration into the infarcted myocardium and conferring cardioprotective effects [27]. Regarding mechanisms, the protective effects of PGRN in MI and I/R injury encompass several intracellular signaling pathways. Experimental evidence shows that PGRN activates the PI3K/Akt signaling pathway, which plays a pivotal role in reducing myocardial fibrosis and improving cardiac repair after I/R injury [27]. PGRN also inhibits the Wnt/β-catenin signaling pathway—a crucial pathway involved in the development of fibrosis [30,31] By suppressing this pathway, PGRN mitigates the extent of fibrosis and adverse cardiac remodeling after ischemic events [32,33]. Specifically, PGRN downregulates Wnt1 expression and β-catenin activation in the heart and kidney, with administration of rPGRN shown to inhibit this signaling cascade [27].

Moreover, PGRN ameliorated the deterioration of cardiac dysfunction and fibrosis by inhibiting Wnt signaling after MI, further preventing adverse cardiac remodeling [27]. Additional **experimental data** in models of endotoxin-induced acute kidney injury (AKI) show that PGRN deficiency exacerbates renal injury, including increased BUN/SCr, higher tubular damage scores, elevated apoptosis (as indicated by TUNEL staining, altered Bax/Bcl-2, and increased cleaved caspase 3), and heightened inflammatory responses [34]. Conversely, rPGRN administration provides significant protection by reducing apoptosis and inflammation as well as inhibiting HMGB1 translocation. These benefits are observed in both wild-type and PGRN-deficient mice, with rPGRN mitigating the exaggerated injury seen in the latter [34]. In cardiac I/R models, rPGRN treatment significantly reduces myocardial infarction size and fibrosis, while inhibiting neutrophil infiltration and inflammation. Mechanistically, PGRN’s protective effects against I/R injury are attributed to its capacity to inhibit neutrophil recruitment, nuclear factor kappa-light-chain-enhancer of activated B cells (NF-κB) pathway activation, and local inflammation [35].

In summary, while clinical studies suggest that elevated serum PGRN is a marker of worse cardiovascular risk in acute MI, extensive experimental evidence demonstrates a cardioprotective and anti-inflammatory role for PGRN in the context of MI and I/R injury, mediated by multiple intracellular signaling pathways and modulation of both inflammatory and fibrotic responses.

#### 3.1.2. Sepsis-Induced and Diabetes-Induced Cardiomyopathy

Sepsis-induced cardiomyopathy (SIC) is a form of cardiac dysfunction that occurs in response to sepsis, characterized by a reversible reduction in cardiac output and ejection fraction [36]. The pathogenesis of SIC is complex and involves a combination of inflammatory responses, mitochondrial dysfunction, and myocardial cell death [37]. PGRN has been shown to play a cardioprotective role in SIC through its anti-inflammatory, anti-atherogenic, and anti-oxidant effects, which are associated with increased nitric oxide synthesis [38,39]. A patient cohort has shown clinical significance of PGRN levels in terms of the severity and prognosis of sepsis [40,41]. Studies have mentioned that PGRN provides a protective effect by regulating macrophage polarization and recruitment, crucial for host defense during sepsis [42].

PGRN plays a pivotal role at the intersection of hormonal, endocrine, and immune pathways, which are central to cardio-protection and systemic metabolic homeostasis. Quantitatively, PGRN levels are significantly elevated in metabolic and endocrine disorders; for example, in a cross-sectional study of 244 obese individuals, increased PGRN concentrations were directly correlated with bone formation parameters and central obesity, implying a protective hormone-sensitive role in bone metabolism [43]. In type 2 diabetes mellitus, circulating PGRN increases and is reduced by lifestyle interventions, with experimental data showing that Grn−/− mice are protected from diet-induced insulin resistance, underscoring PGRN’s involvement in endocrine regulation via the TNFR1–IL-6 inflammatory pathway [44]. Additionally, PGRN modulates cartilage and bone anabolism through TNFR2 activation—a receptor involved in anti-inflammatory and regenerative endocrine signaling—and this pathway is quantified as having a 600-fold higher PGRN binding affinity to TNFR2 than TNF-α, emphasizing the specific and powerful endocrine receptor interactions [45]. Moreover, in cardiovascular terms, PGRN augments endothelium-dependent relaxation by enhancing nitric oxide signaling and cGMP production, and its level is positively correlated with body mass index, cholesterol, and glucose [46]. These findings demonstrate that PGRN is not only an immunomodulator but also directly interfaces with hormonal and endocrine regulators, confirming its centrality in the complex, intermingled mechanisms underlying cardio-protection and related endocrine diseases.

PGRN significantly relates with the high mortality rates associated with sepsis [40]. Research has shown that PGRN-deficient mice exhibited increased mortality in models of polymicrobial sepsis and endotoxemia due to heightened tissue levels of inflammatory cytokines and reduced IL-10 production [47]. Administration of recombinant PGRN decreased susceptibility to endotoxic shock and enhanced IL-10 production by macrophages in a TNFR-dependent manner. In this mechanism, PGRN interacted with C/EBPα and stabilizes it to promote the production of IL-10. PGRN targeting the ubiquitin–proteasome system to preserve C/EBPα stability represents another promising strategy. Another study investigated the impact of alpha-lipoic acid (ALA) treatment on serum PGRN levels and inflammatory markers in patients with type 2 diabetes and peripheral neuropathy [48]. Fifty-four patients received 600 mg of ALA daily for six months. Results showed significant increases in PGRN levels and improvements in sensory neuropathy. Correlations between PGRN and inflammatory markers, like tumor necrosis factor-alpha (TNFα) and ICAM-1, were observed. The study concluded that ALA treatment may improve endothelial function and reduce neuronal inflammation via PGRN in diabetic neuropathy patients.

#### 3.1.3. Cardiac Aging and Hypertrophy

Cardiac aging is a complex pathophysiological process characterized by structural and functional changes in the heart, including left ventricular hypertrophy, diastolic dysfunction, and alterations in heart rhythms [49,50]. PGRN is a critical protein involved in numerous cellular processes, including inflammation regulation, lysosomal function, and cellular repair [51,52]. Serum PGRN levels may be a candidate biomarker for sarcopenia-related physical frailty, highlighting the potential role of PGRN in this condition [53]. Specifically, PGRN deficiency has been shown to accelerate age-related cardiac abnormalities, such as ventricular hypertrophy and cardiac dysfunction [54]. In animal models, PGRN knockout (KO) mice exhibited more pronounced signs of cardiac aging compared to wild-type [4]. PGRN maintains cardiac health by controlling inflammatory responses and preserving cellular homeostasis.

The mechanisms through which PGRN influences cardiac aging are multifaceted. PGRN is involved the Wnt/β-catenin signaling pathway, which is activated by the binding of complement component 1q (C1q) to cell surface receptors [54]. PGRN deficiency leads to increased C1q expression, which subsequently activates β-catenin, promoting cardiomyocyte hypertrophy and cardiac dysfunction [54]. In addition, PGRN is integral to lysosomal function and protein homeostasis. PGRN absence resulted in impaired lysosomal acidification and protein degradation, contributing to the accumulation of damaged proteins and cellular debris, thereby accelerating cardiac aging [55]. These mechanisms highlight the essential role of PGRN in maintaining cardiac function and mitigating the adverse effects of aging on the heart.

PGRN serves as a critical mediator in both skeletal muscle hypertrophy and cardiac hypertrophy, acting through distinct cellular and molecular mechanisms to support tissue adaptation and growth. In skeletal muscle, recent single-cell RNA-seq and genetic depletion studies have demonstrated that PGRN is predominantly produced by macrophages (Mø) in response to mechanical overload. PGRN orchestrates a relay of communication among mesenchymal progenitors, infiltrating macrophages, and muscle satellite cells (MuSCs), facilitating efficient muscle hypertrophy by supporting MuSC proliferation and differentiation. This process enhances myonuclear accretion and myofiber growth, essential for physiological hypertrophy [56]. Furthermore, the absence of PGRN impairs these intercellular signals, resulting in blunted muscle growth. Interestingly, in the context of muscle regeneration, PGRN deficiency leads to persistence of anti-inflammatory (M2) macrophages, which correlates with augmented myofiber hypertrophy but also carries a risk of excessive fibrosis due to aberrant repair [57]. In cardiac hypertrophy and pathological settings such as hyper-homo-cysteinemia-induced cardiorenal injury, PGRN confers protection by negatively regulating the Wnt/β-catenin signaling pathway—a pathway implicated in hypertrophic and fibrotic remodeling of the heart. Experimental models showed that PGRN supplementation attenuates organ hypertrophy, inflammation, and fibrosis, suggesting therapeutic potential in cardiovascular diseases [32]. In summary, PGRN plays a dual role in regulating both skeletal muscle and cardiac hypertrophy, acting through coordination of inflammatory cell clearance, modulation of myogenic cell fate, and inhibition of pro-fibrotic signaling pathways. These findings highlight the promise of PGRN as a therapeutic target in both physiological adaptation and pathological hypertrophy.

PGRN significantly modulates muscle hypertrophy and function through specific cellular mechanisms and quantifiable effects. In mammalian muscle cells, treatment with 500 ng/mL PGRN for 48 h increased C2C12 (murine myoblast cells derived from satellite cells) myotube diameter and fusion index by approximately 35% and 30%, respectively, compared to controls, exhibiting efficiency nearly equivalent to that of IGF-1 [58]. PGRN-induced hypertrophy depends on the PI3K/Akt/mTOR pathway, as pharmacological inhibition with LY294002 (20 µM) or rapamycin (100 nM) abolishes the hypertrophic effects and phosphorylation of downstream targets Akt, GSK-3α/β, and p70S6K. Furthermore, when IGF-1 signaling was blocked using 30 µM PQ401, PGRN supplementation fully rescued the atrophic phenotype, restoring both fusion index and nuclei per myotube to levels not statistically different from untreated controls. Complementarily, in zebrafish models, knockdown of the PGRN ortholog GRN A reduced the number of paired box 7 positive myogenic progenitor cells by over 66% and decreased myofiber cross-sectional area by 39% at 6 dpf, while muscle-specific overexpression of GRN A increased activated progenitors by 1.9-fold after injury and yielded a 35% larger myofiber area in juveniles [59]. Mechanistically, PGRN preserves muscle growth and function by maintaining the progenitor pool, suppressing apoptosis (with a 5.1-fold increase in apoptotic cells in PGRN-deficient embryos), and triggering hypertrophic signaling through MET and PI3K/Akt/mTOR cascades, positioning it as an essential factor in muscle development and regeneration. Furthermore, the interaction of PGRN with TNFR is crucial for regulating cardiac hypertrophy and other cardiovascular complications [17]. PGRN directly interacting with TNFR and significantly enhanced the Treg positive population and stimulated IL-10 production [19]. PGRN regulates cardiac aging and muscle atrophy through its involvement in inflammation control, lysosomal function, and cellular repair, while also showing potential as a biomarker for sarcopenia-related physical frailty.

**Figure 2:**
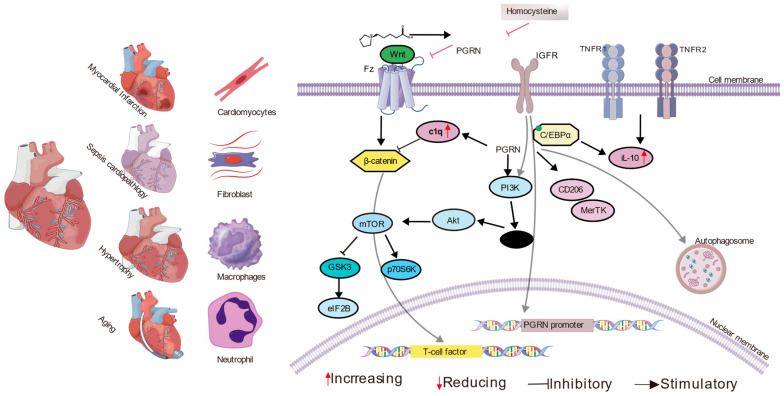
The multifaceted role of Progranulin (PGRN) in various cardiac pathologies. This diagram illustrates how PGRN is involved in different cardiac conditions, including myocardial infarction and ischemia-reperfusion injury, sepsis-induced cardiomyopathy, and cardiac aging and hypertrophy. The figure depicts various cell types involved in these processes, such as cardiomyocytes, fibroblasts, macrophages, and neutrophils. Key molecular interactions are shown, including PGRN binding to receptors like SORT1, TNFR1, and TNFR2, and its influence on other molecules, like C1q and components of the Wnt pathway. The diagram outlines several intracellular signaling cascades affected by PGRN, such as the activation of the PI3K/Akt and mTOR pathways and the inhibition of the Wnt/β-catenin and NF-kappa B pathways, which collectively modulate inflammation, fibrosis, apoptosis, and cellular homeostasis. Additionally, the regulation of the PGRN promoter by the IGF-1 receptor is indicated. These mechanisms collectively underscore PGRN’s protective and modulatory effects in the context of diverse cardiac diseases.

Together, we selected cardiac pathologies for which original research provides direct mechanistic and functional data on PGRN’s impact. Studies on PGRN’s role in established heart failure remain limited, which we identify as a key area for future investigation. PGRN is a critical factor that protects against myocardial infarction, sepsis-induced cardiomyopathy, and cardiac aging by reducing inflammation, fibrosis, and adverse remodeling, while also enhancing macrophage function and maintaining cellular homeostasis, as shown in Figure 2.

### 3.2. Mechanisms of Progranulin’s Impact on Vascular Diseases

#### 3.2.1. Abnormal Angiogenesis or Vascular Injury

Dysfunction of PGRN has emerged as a significant risk factor for the development of CVD, positioning PGRN as a potential therapeutic target for high-risk populations. PGRN plays a critical role in maintaining vascular smooth muscle cell (VSMC) contractility, metabolic activity, and oxidative stress control [60,61].

Research indicates that PGRN is intimately related to angiogenesis, particularly during the formation of the placental labyrinth. PGRN KO mice have demonstrated substantial impairments in placental vascular development. These PGRN KO mice exhibited reduced placental weight and lower pup body weight compared to their wild-type counterparts. Notably, the labyrinthine layer area was significantly diminished, indicating abnormal vascularization. Vascular markers, such as α-smooth muscle actin (SMA) and CD31, showed a reduction of 40% in PGRN KO placentas at embryonic days 14.5 and 17.5. Furthermore, expression levels of endothelial nitric oxide synthase (eNOS) were decreased by 50%, leading to compromised vasodilation [62].

Additional research has highlighted the potential role of the PGRN/EphrinA2 (EphA2) axis in mediating homocysteine (HCY)-induced endothelial adhesion and vascular injury, with implications for endothelial cell barrier integrity [63]. Elevated levels of homocysteine, recognized as a risk factor for cardiovascular diseases, modulate the expression of PGRN and EphA2 in endothelial cells. Knockdown of EphA2 significantly impaired cell adhesion, migration, and angiogenesis—all processes critical for effective vascular repair. Notably, PGRN interaction has been shown to ameliorate these effects by restoring cell viability and migration, primarily through the PGRN/EphA2 axis and its downstream AKT/NF-κB signaling pathways. Clinical investigations involving 201 patients revealed that both PGRN and EphA2 levels were significantly elevated in individuals with acute coronary syndrome (ACS) compared to those with stable angina pectoris (SAP) or healthy control subjects [64]. These findings suggest that measuring circulating levels of PGRN and EphA2 could facilitate early diagnosis and prognosis of coronary artery disease (CAD), particularly in assessing the severity of atherosclerosis and the risk of acute cardiovascular events.

Another study identified PGRN as an essential regulator of vascular homeostasis factor. Deficiency of PGRN markedly impairs mitochondrial function in vascular smooth muscle cells (VSMC), evidenced by significant reductions in mitochondrial oxygen consumption rates and complex I enzyme activity, as well as a notable increase in the accumulation of mitophagy and autophagy impairment markers, such as p62, LC3I/II, PINK, and PARKIN—up to 2- to 3-fold higher than controls. Functional consequences include a robust defect in vascular contractility and heightened susceptibility to vascular injury; for example, loss of PGRN decreases aortic contractile responses to KCl, phenylephrine, and thromboxane A2 analog by more than 30% compared to wild type, and results in greater vascular fibrosis following Angiotensin II stress [60,61]. Importantly, overexpression of PGRN, or restoration via lentiviral delivery or treatment with the autophagy-inducing agent spermidine, was able to rescue mitochondrial respiration (increasing oxygen consumption rates to near-normal levels) and restore contractile function in PGRN-deficient mice. These quantitative data collectively demonstrate that PGRN is indispensable for maintaining mitochondrial quality and proper mitophagic flux, thus preventing abnormal angiogenesis, vascular dysfunction, and injury. This underscores PGRN’s therapeutic potential in vascular diseases characterized by impaired mitochondrial homeostasis and regenerative capacity.

The absence of PGRN is linked to significant vascular impairments, including elevated mean arterial pressure (MAP) and reduced contractility of vascular smooth muscle cells (VSMCs), which can ultimately contribute to abnormal angiogenesis and vascular injury [65,66]. Restoration of PGRN levels through administration of rPGRN via osmotic minipump for 7 days demonstrated substantial therapeutic potential. In PGRN KO mice treated with rPGRN, this approach reversed the observed vascular dysfunction and restored vascular tone and contractility, as well as recovering mean arterial pressure. Notably, the protective effects of PGRN, such as attenuating vascular contractility, are mediated through its interaction with the EphrinA2 receptor and Sortilin1. This mediation involves the activation of endothelial nitric oxide synthase (eNOS) and the production of nitric oxide (NO) in endothelial cells, which is crucial for regulating vascular tone. The abolishment of rPGRN’s anticontractile effect and its ability to activate eNOS/produce NO when EphrinA2 or Sortilin1 are pharmacologically blocked. In the other study, PGRN is identified as a key protein in maintaining vascular health, particularly affecting vascular smooth muscle cell (VSMC) contractility in conductance arteries, like the aorta [67]. The mechanisms described link PGRN’s function critically to mitochondrial quality and cellular bioenergetics. Specifically, PGRN is shown to preserve mitochondrial balance through modulating mtROS signaling, influencing mitochondrial function and dynamics (fission and fusion), and regulating mitophagy flux, the process responsible for clearing damaged mitochondria. Deficiency in PGRN leads to impaired mitophagy flux, mitochondrial fragmentation, decreased expression and activity of mitochondrial complex I, a disrupted NAD+/NADH ratio, diminished ATP levels, and excessive mtROS generation, all of which compromise mitochondrial quality and result in loss of VSMC contractility. Furthermore, PGRN regulates lysosome biogenesis in a TFEB-dependent manner and lysosome function, processes intertwined with mitochondrial health and autophagy. Thus, the PGRN level maintains vascular contractility and prevents functional decline by managing mitochondrial quality via the regulation of lysosome biogenesis, mitophagy, and complex I pathways.

Endothelial cells demonstrate a notable responsiveness to PGRN, as evidenced by the phosphorylation of endothelial nitric oxide synthase (eNOS) and the resulting elevation in nitric oxide production following PGRN treatment [65,68]. PGRN deficiency has been associated with heightened levels of inflammatory markers and increased sodium excretion; specifically, PGRN knockout (KO) mice exhibited a two-fold increase in circulating CCL2 levels and a 1.5-fold increase in sodium excretion. Such mitochondrial dysfunctions play a critical role in the pathogenesis of CVD, as they compromise vascular integrity and foster an inflammatory environment. Given these findings, targeting the PGRN pathway emerges as a promising strategy for therapeutic interventions aimed at alleviating vascular injury and restoring normal angiogenic processes, offering potential benefits for individuals at risk for or affected by CVD.

#### 3.2.2. Atherosclerosis

In the initial stages of human atherosclerotic plaque development, PGRN is expressed in smooth muscle cells (SMCs) and macrophages, promoting TNFα-mediated SMC migration [69]. As atherosclerosis advances, PGRN plays a protective role by inhibiting plaque formation through the suppression of monocyte chemoattractant protein-1 (MCP-1)-induced THP-1 cell migration [70,71]. Additionally, as a regulator of inflammation, PGRN reduces interleukin-8 (IL-8) secretion in human aortic smooth muscle cells (HASMCs) [70]. However, the cleavage products of PGRN, known as granulin peptides (GRNs), are implicated in exacerbating inflammation by facilitating the recruitment of neutrophils and monocytes, thereby promoting the progression of atherosclerosis [70,72,73].

PGRN is a multifunctional glycoprotein that has emerged as an important factor in the development of atherosclerosis [57,69]. It is highly expressed in macrophages and foam cells within atherosclerotic plaques, suggesting a role in local vascular pathophysiology [69]. Mechanistically, PGRN exerts athero-protective effects through multiple pathways. It suppresses inflammation by downregulating the expression of proinflammatory cytokines, such as TNF-α and IL-1β, as well as adhesion molecules, including ICAM-1 and VCAM-1, thereby limiting leukocyte recruitment and vascular inflammation. PGRN also preserves endothelial function via maintenance of endothelial nitric oxide synthase (eNOS) levels, promoting vascular homeostasis. At the cellular level, PGRN modulates cholesterol metabolism in macrophages by enhancing the expression of cholesterol efflux transporters (e.g., ABCA1, ABCG1) and reducing the expression of cholesterol uptake receptors (e.g., CD36, scavenger receptor A), thereby limiting foam cell formation. Additional studies have shown that deficiency of PGRN leads to increased exophagy-mediated uptake and storage of cholesterol from aggregated LDL in macrophages, further promoting foam cell formation and atherogenesis [57]. In mouse models, genetic deletion of PGRN in an atherogenic background (such as ApoE or LDLr knockout) accelerates atherosclerosis despite a favorable plasma lipid profile, underscoring the importance of PGRN’s local actions in the vessel wall rather than systemic lipid modulation. Collectively, these findings indicate that PGRN acts as a protective factor in atherosclerosis, primarily by suppressing vascular inflammation, supporting endothelial integrity, and maintaining intracellular cholesterol homeostasis in macrophages.

Moreover, circulating PGRN has been found to exhibit limited capacity to suppress accelerated atherosclerosis. PGRN possesses notable antiplatelet and anticoagulant properties, demonstrated in a study where it significantly inhibited platelet aggregation and prolonged bleeding time, prothrombin time, and activated partial thromboplastin time in a dose-dependent manner in rat models [74]. In vitro investigations revealed that PGRN significantly inhibits platelet aggregation in response to adenosine diphosphate (ADP) and arachidonic acid (AA), underscoring its powerful antiplatelet activity. These findings suggest that PGRN can inhibit both intrinsic and extrinsic pathways of the coagulation cascade, reinforcing its physiological protective role against thrombotic disorders commonly associated with atherosclerosis.

### 3.3. Calcific Aortic Valve Disease

**Figure 3:**
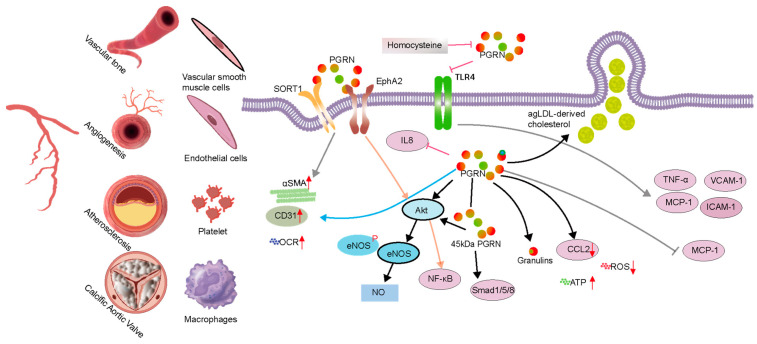
Progranulin (PGRN) in vascular pathophysiology. PGRN regulates critical cellular constituents involved in vascular health and disease, including vascular smooth muscle cells, endothelial cells, platelets, macrophages, and calcific aortic valve cells. The figure illustrates that PGRN interacts with multiple receptors, notably SORT1 and EphA2, initiating diverse downstream signaling cascades. In endothelial cells, PGRN activates the Akt/eNOS/NO pathway, which promotes vasodilation and contributes to blood pressure regulation. Concurrently, PGRN modulates inflammatory responses by attenuating the expression of adhesion molecules and pro-inflammatory cytokines, including VCAM-1, ICAM-1, TNF-α, and MCP-1, primarily through the inhibition of the NF-κB pathway.

Recent studies have unveiled novel mechanisms contributing to the pathogenesis of calcific aortic valve disease (CAVD) and have highlighted the potential role of PGRN as a mediator of valve fibrosis and osteoblastic differentiation [75]. In human valve interstitial cells (hVICs) subjected to calcifying conditions, there was a marked increase in the expression of the 45-kDa degradation product of PGRN, known as 45kd-PGRN, accompanied by a concurrent reduction in total PGRN levels. Notably, the exogenous addition of 45kd-PGRN was found to enhance calcification in hVICs by activating key signaling pathways, including Smad1/5/8, NF-κB, and AKT. In contrast, inhibition of these pathways led to a significant attenuation of 45kd-PGRN’s effects on the transition of the VIC phenotype, underscoring the importance of these signaling cascades in mediating valve pathology. Furthermore, the study revealed that 45kd-PGRN displays contrasting expression patterns compared to PGRN in response to stimulation with tumor necrosis factor-alpha (TNFα), indicating a complex and nuanced role for PGRN in the modulation of CAVD progression. Collectively, these findings suggest that PGRN is a critical protein regulating vascular function, angiogenesis, atherosclerosis, and metabolic disorders, where its deficiency contributes to impaired vascular health, altered lipid metabolism, and an elevated risk of cardiovascular diseases, as illustrated in Figure 3. This emerging understanding of PGRN’s multifaceted role in CAVD opens new avenues for potential therapeutic interventions targeting this critical pathway.

### 3.4. Progranulin’s Role in Cardiovascular Metabolism and Metabolic Disorders

Metabolic diseases, such as diabetes and obesity, significantly increase the risk of CVD, including heart disease and stroke, by causing lipid abnormalities, hypertension, and chronic inflammation [76]. Emerging evidence indicates that PGRN may function as a regulatory factor in the intricate interplay between metabolic and cardiovascular processes [77]. Notably, studies investigating nutrient metabolism have revealed that PGRN deficiency leads to an increased preference for high-fat foods, which subsequently alters daily intake patterns [78]. PGRN-deficient mice exhibited a distinct inclination toward higher-fat options—favoring 2% milk over 0.3% milk and medium-chain triglycerides over water—resulting in a significant 10% body weight gain over a seven-day observation period. This altered feeding behavior was linked to reduced expression of the CD36 receptor in taste buds, which is critical for fat taste perception; diminished CD36 levels essentially lowered the sensory response to dietary fats, driving the increased consumption of fatty foods for sensory satisfaction.

In hypercholesterolemic models, significant differences in PGRN levels were observed between patients with familial hypercholesterolemia and healthy controls [79]. Noteworthy positive correlations were established between serum PGRN levels and various metabolic markers, including triglycerides, tumor necrosis factor-alpha (TNFα), soluble vascular cell adhesion molecule-1 (sVCAM-1), and sHDL subfractions [79]. PGRN, secreted by human monocyte-derived macrophages, was found to associate with apolipoprotein A-I (apoA-I). This association suggests a potential role in athero-protection by suppressing the pro-inflammatory properties of progranulin. The study found that HDL/apoA-I binding to progranulin inhibits its conversion into pro-inflammatory granulins, which are known to stimulate inflammatory cytokine production (like TNF-alpha and IL-1beta) [80]. In cellular studies, unesterified cholesterol and cholesteryl ester levels remained consistent across control, PGRN-knockout, and PGRN-knockin cells, in contrast to altered cholesterol levels in NPC1- or NPC2-knockout cells, which are deficient in cholesterol export from lysosomes [81]. Furthermore, bone marrow transplantation in PGRN-deficient mice, conditioned with busulfan and PLX3397, successfully restored PGRN levels in the brain and eyes, normalizing brain lipofuscin storage, proteo-stasis, and lipid metabolism [57,82].

Another pivotal study assessed serum concentrations of PGRN and FAM19A5 in patients with metabolic syndrome (MS) compared to healthy individuals [83]. Correlations noted in MS patients suggested that FAM19A5 levels negatively correlated with diastolic blood pressure and cholesterol, pointing towards a potential role in dyslipidemia and hypertension. These observations necessitate further investigation into the underlying mechanisms and therapeutic implications.

PGRN also regulates mitochondrial respiration by impacting complex I activity and maintaining mitochondrial dynamics [60,84]. PGRN deficiency has been associated with mitochondrial fragmentation, resulting in decreased ATP production and increased reactive oxygen species (ROS) generation. Disruption in mitophagy due to PGRN absence leads to the accumulation of dysfunctional mitochondria, further contributing to the pathogenesis of CVD. As a pivotal protein in cardiovascular metabolism, PGRN mediates the cross-talk between immune responses and lipid turnover through its dual immunomodulatory and lipoprotein-like properties.

Moreover, research has shown that the PGRN-sirtuin1 signaling pathway critically regulates the activity of the transcription factor FoxO1, which plays a crucial role in cardiovascular metabolism by influencing aging, cellular metabolism, insulin resistance, and oxidative stress resistance [7]. In high-glucose-treated cells, PGRN decreased FoxO1 acetylation by sirtuin1, highlighting its involvement in metabolic regulation through both direct and indirect mechanisms, including modulation of phosphorylation and acetylation levels of FoxO1 and inhibition of sirtuin expression. These findings underscore the multifaceted role of PGRN in cardiovascular metabolism and its potential as a therapeutic target for metabolic disorders and associated cardiovascular complications.

### 3.5. PGRN as an Autophagosome and Lysosome Regulator

Autophagosomes and lysosomes are essential for cardiovascular health, functioning to degrade and recycle long-lived proteins and organelles, thereby maintaining cardiac homeostasis [85]. Dysregulated autophagy, whether reduced or excessive, has been associated with heart failure and aging, marking it as a potential therapeutic target for cardiovascular diseases. PGRN plays a critical role in maintaining lysosomal function and autophagic flux. Recent studies have demonstrated that PGRN deficiency leads to the accumulation of autophagic substrates, such as p62 and LC3II, serving as indicators of impaired autophagy, through its regulation of transcription factor EB (TFEB), which promotes lysosomal gene expression [16,55,86,87]. The absence of PGRN results in dysfunctional lysosomes characterized by abnormal accumulation of lysosomal-associated membrane proteins (LAMPs) and increased nuclear TFEB levels, despite an increase in lysosome formation [88].

PGRN has been shown to be essential for lysosomal function, as evidenced by research conducted by Doyle et al., which revealed that PGRN deficiency leads to significant lysosomal dysfunction [89]. This dysfunction is characterized by altered lysosomal morphology, increased lysosomal biogenesis, and the presence of smaller lysosomes exhibiting heightened fluorescence intensity of lysosomal membrane protein 1 (LAMP-1), indicative of substantial changes in lysosomal content and functionality. The impact of this dysfunction extends to the autophagic process, which relies on lysosomes for the degradation and recycling of cellular components. In PGRN-deficient *Caenorhabditis elegans* models, decreased puncta of LGG-1 (the LC3 homolog) indicated a marked reduction in autophagosome formation. PGRN deficiency disrupts the conversion of autophagosomes into autolysosomes, leading to an accumulation of autophagosomes and a concurrent reduction in autolysosome formation, which ultimately impairs autophagic flux. These findings underscore the critical role of PGRN in maintaining both lysosomal integrity and autophagic activity.

Additionally, PGRN deficiency disrupts the fusion process between autophagosomes and lysosomes, resulting in the accumulation of autophagosomes and impaired lysosomal degradation. This dysfunction increases the secretion of autophagy-associated vesicles containing pathological proteins such as TDP-43 [90]. Inhibition of vacuolar ATPase (v-ATPase)—a key enzyme essential for lysosomal acidification—has been shown to enhance TDP-43 secretion via extracellular vesicles, a process that mimics the effects observed in PGRN deficiency. The absence of PGRN leads to increased secretion of cleaved TDP-43 through extracellular vesicles, facilitating the dissemination of TDP-43 pathology in neurodegenerative diseases. This secretion is autophagy-dependent, as reductions in TDP-43 secretion have been recorded in autophagy-deficient cellular models. Consequently, dysregulation of PGRN-related pathways plays a significant role in the propagation of TDP-43 pathology, emphasizing the therapeutic potential of targeting these mechanisms to mitigate neurodegenerative progression.

PGRN also critically regulates autophagosome and lysosome functions, maintaining lysosomal integrity by modulating the activity of key enzymes, such as cathepsins and glucocerebrosidase (GCase) [91]. PGRN stabilizes these enzymes and influences levels of bis(mono-acyl-glycero)phosphate (BMP), which are vital for lysosomal lipid degradation [81]. Deficiency in PGRN results in decreased BMP levels, impaired GCase activity, and subsequent lysosomal dysfunction, contributing to the development of neurodegenerative diseases. Moreover, PGRN acts as a non-redundant regulator of systemic inflammation by modulating the levels and activity of C/EBPα and IL-10 through the E6-AP-mediated ubiquitin–proteasome proteolysis pathway [47].

**Figure 4:**
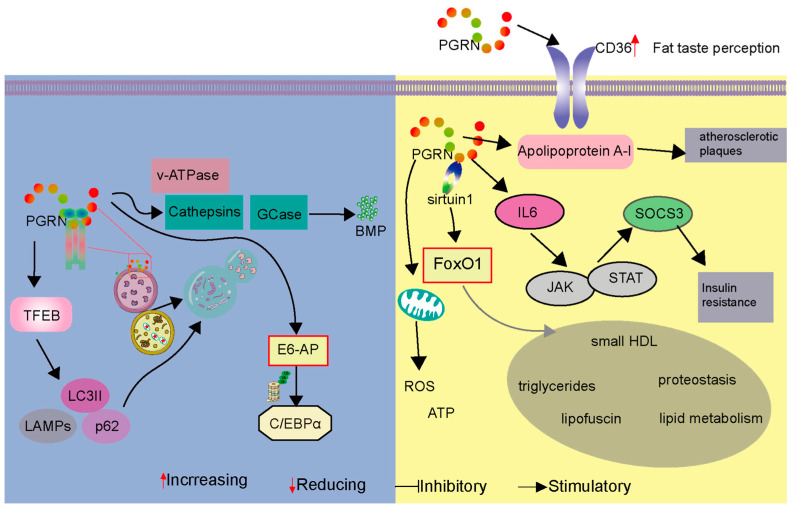
PGRN’s multifaceted roles in lysosomal function, autophagy, and metabolism impacting cardiovascular health. The figure is divided into two panels illustrating distinct but related functions of Progranulin (PGRN). The Left panel depicts PGRN’s influence on lysosomal function and autophagy. PGRN regulates essential lysosomal enzymes, like cathepsins and glucocerebrosidase (GCase), and influences bis(mono-acyl-glycero)phosphate (BMP) levels, all critical for maintaining lysosomal integrity and function. PGRN deficiency is shown to lead to the accumulation of autophagosomes, indicated by increased levels of p62 and LC3-II, and causes alterations in lysosomal-associated membrane protein (LAMP) dynamics, reflecting impaired lysosomal degradation and autophagic flux. The E6-AP mediated ubiquitin–proteasome pathway is also illustrated, modulating C/EBPα levels, which affects inflammatory responses. The Right panel focuses on PGRN’s impact on metabolic and cardiovascular processes. PGRN is shown to interact with CD36, a receptor involved in fat taste perception, suggesting a role in regulating feeding behavior. It associates with apolipoprotein A-I (apoA-I), primarily within the context of HDL, influencing HDL metabolism. This association is described in the text as contributing to athero-protection by inhibiting the conversion of PGRN into pro-inflammatory granulins. While the figure connects this interaction to “atherosclerotic plaques”, the text clarifies that PGRN within plaques (expressed by SMCs, macrophages, and foam cells) exerts protective effects by suppressing inflammation, modulating cholesterol metabolism, and inhibiting plaque progression, rather than directly causing or stabilizing plaque structure. The PGRN-sirtuin1 signaling pathway is depicted regulating FoxO1 activity, which is crucial for cardiovascular metabolism, aging, insulin resistance, and oxidative stress. Furthermore, PGRN’s modulation of IL-6 production and the JAK-STAT pathway is shown to lead to SOCS3 expression and subsequent insulin resistance. The panel also indicates PGRN’s influence on mitochondrial function, affecting ATP production and ROS levels. This dual depiction highlights PGRN’s essential contributions to maintaining cellular homeostasis, regulating metabolic processes, and supporting cardiovascular health through diverse mechanisms.

PGRN is vital for the maturation of autophagosomes and their fusion with lysosomes; its deficiency induces autophagic defects that further exacerbate neurodegenerative processes. Therapeutic strategies aimed at restoring PGRN function hold promise for treating these diseases, highlighting its potential as a valuable therapeutic target. PGRN regulates lysosomal function and autophagy (left panel of Figure 4) while also modulating metabolic processes and insulin resistance through interactions with sirtuin1, FoxO1, and apolipoprotein A-I (right panel of Figure 4). This dual role emphasizes PGRN’s essential contributions to maintaining cellular homeostasis and supporting cardiovascular health.

## 4. Therapeutic Approach Targeting PGRN

Recent advancements in strategies aimed at augmenting PGRN levels have demonstrated considerable therapeutic potential for addressing various pathological conditions. Four primary approaches have emerged to enhance PGRN levels. Firstly, direct protein replacement therapy has gained prominence, exemplified by the innovative use of DNL593 [92]. This candidate utilizes protein transport vehicle (PTV) technology to facilitate PGRN delivery across the blood–brain barrier into the central nervous system (CNS). A seminal study published in Cell by researchers at Denali Therapeutics elucidated the development of this PTV:PGRN (DNL593) approach, shedding light on the pathogenesis of frontotemporal dementia (FTD) and lysosomal function in of PGRN deficiencies [93]. The efficacy of DNL593 in treating FTD due to PGRN deficiency is currently under evaluation in a phase I/II clinical trial (NCT05262023) conducted by Denali Therapeutics in collaboration with Takeda [94]. This placebo-controlled study involves 106 participants across 14 sites in Europe, Turkey, and Brazil, with the aim of assessing safety, tolerability, and pharmacokinetics during November 2025. A summary of the advancements in clinical trials is provided in Table A1.

### 4.1. Protein Replacement

Concurrently, several indirect methods to induce PGRN overexpression have been developed using various vectors. Adeno-associated viruses (AAVs) have proven instrumental in gene therapy, given their ability to deliver genetic material to specific cells and tissues. One notable example is the PR006 (LY3884963) gene-replacement therapy, which employs AAV9 to deliver a functional copy of the PGRN gene (GRN) to the brain, targeting mutations responsible for FTD [95]. This therapy aims to restore normal PGRN levels, thereby improving lysosomal function and reducing neuroinflammation. Initial results from a Phase 1/2 trial initiated in 2020 have shown promising increases in cerebrospinal fluid (CSF) PGRN levels, although some patients experienced adverse events, including neuroinflammation and blood clots. Another preclinical initiative, PBFT02, is comparing the efficacy of different AAV serotypes (AAV1, AAV5, and AAVhu68) in delivering the GRN gene to achieve therapeutic PGRN levels in the CNS [96,97,98]. Notably, evaluated AAV vector administration into the cerebrospinal fluid of GRN-deficient mice and nonhuman primates (NHPs) resulted in the normalization of lysosomal function, as evidenced by restored hexosaminidase activity and a reduction in lipofuscin accumulation in the brain, both indicative of compromised lysosomal integrity. Notably, evaluated AAV vector administration into the cerebrospinal fluid of GRN-deficient mice and nonhuman primates (NHPs) resulted in the normalization of lysosomal function, as evidenced by restored hexosaminidase activity and a reduction in lipofuscin accumulation in the brain, both indicative of compromised lysosomal integrity. The PBFT02 clinical trial is investigating a gene therapy that uses an AAV1 vector to deliver the GRN gene directly to the brain [99]. Early results from the Phase 1/2 UpliFT-D trial showed that the therapy effectively increased cerebrospinal fluid (CSF) PGRN levels by 3.6 to 6.6 times above baseline, indicating successful delivery and expression. While some adverse events, such as liver toxicity and deep vein thrombosis, were reported, the therapy was generally well-tolerated, with no significant immune responses or peripheral nerve damage observed.

AVB-101 represents another investigational AAV9 vector-delivered gene therapy targeting neurons to address frontotemporal dementia [100,101,102]. The ongoing Phase 1/2 AVB-101 trial is aimed at evaluating the safety and preliminary efficacy of this approach, with preclinical studies in non-human primates indicating that intra-thalamic delivery can achieve widespread brain expression of PGRN with minimal systemic exposure. Recent efforts have also improved the PTV:PGRN strategy through AAV packaging that targets the liver to produce a transferrin receptor-binding protein fusion PGRN variant (AAV(L):bPGRN), as demonstrated in two animal models [103]. The preclinical research about PGRN overexpression in the cardiovascular environment also can alleviate the disease.

### 4.2. SORT1-Axis Modulation

Another promising strategy for maintaining PGRN levels involves targeting the PGRN–SORT1 axis, which could enhance the secretion of PGRN and underscore its protective roles. Recent studies have identified one antibody and several small molecules that inhibit the interaction between PGRN and SORT1, thereby reducing PGRN degradation. One notable example is Latozinemab (AL001), a human monoclonal antibody that binds specifically to SORT1, preventing its interaction with PGRN, which may increase both plasma and interstitial fluid (ISF) PGRN levels [104]. Research indicated that AL001 elevated PGRN levels in plasma and CSF, potentially slowing disease progression in carriers of heterozygous loss-of-function GRN mutations [105]. Additionally, behavioral deficits associated with PGRN haploinsufficiency were ameliorated.

**Figure 5:**
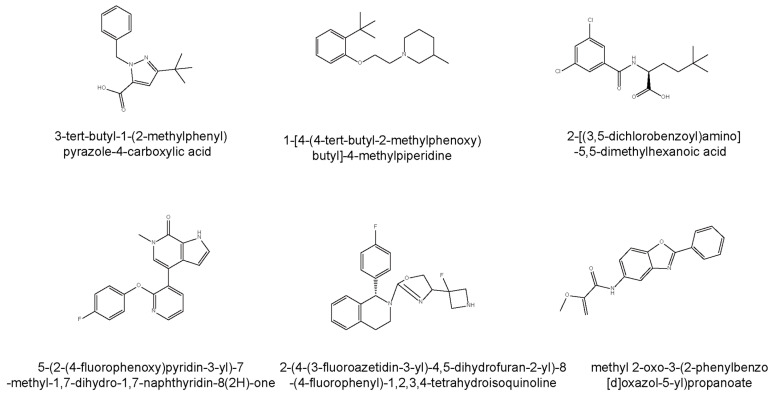
Chemical structures and mechanisms of compounds designed to modulate progranulin (PGRN) levels. This figure illustrates the chemical diversity of compounds developed to influence PGRN levels through two distinct therapeutic strategies. The **top panel** (Compounds **1–3**) presents inhibitors that disrupt the interaction between PGRN and Sortilin 1 (SORT1), thereby increasing extracellular PGRN concentrations. These include **3-tert-butyl-1-(2-methylphenyl)pyrazole-4-carboxylic acid**, **1-[4-(4-tert-butyl-2-methylphenoxy)butyl]-4-methylpiperidine** and **2-[(3,5-dichlorobenzoyl)amino]-5,5-dimethylhexanoic acid**. The **bottom panel** (Compounds **4–6**) displays direct PGRN modulators that enhance PGRN expression or secretion through alternative biochemical pathways. These compounds are **5-(2-(4-fluorophenoxy)pyridin-3-yl)-7-methyl-1,7-dihydro-1,7-naphthyridin-8(2H)-one** (targeting BET domain proteins), **2-(4-(3-fluoroazetidin-3-yl)-4,5-dihydrofuran-2-yl)-8-(4-fluorophenyl)-1,2,3,4-tetrahydroisoquinoline** (promoting PGRN secretion), **methyl 2-oxo-3-(2-phenylbenzo[d]oxazol-5-yl)propanoate** (upregulating PGRN protein levels).

Furthermore, there are two kinds of ligands to regulate the level of PGRN, labeled **1**–**6** in Figure 5. The first class comprises inhibitors that disrupt the interaction between PGRN and SORT1, leading to increased extracellular PGRN levels. Compounds such as **3-tert-butyl-1-(2-methylphenyl)yrazole-4-carboxylic acid** (with an IC50 of 2 μM) and **1-[4-(4-tert-butyl-2-methylphenoxy)butyl]-4-methylpiperidine** not only inhibited SORT1 but also promoted the secretion of PGRN in mammalian cells [14,99,106]. **2-[(3,5-dichlorobenzoyl)amino]-5,5-dimethylhexanoic acid**, which demonstrated a significantly lower IC50 of 0.17 μM, is a potent inhibitor, making it particularly valuable for studying diseases reliant on PGRN modulation. Mechanistically, **2-[(3,5-dichlorobenzoyl)amino]-5,5-dimethylhexanoic acid** anchors via a salt bridge with ARG292, alongside further interactions with SORT1 residues SER283 and TYR318, while its structure encompasses dynamic interactions that account for the observed promiscuity and the flat structure-activity relationship characteristic of this region [106].

### 4.3. Allosteric Activators

The second class of therapeutic candidates seeks to enhance PGRN expression or secretion through different biochemical pathways. Modulators, such as **5-(2-(4-fluorophenoxy)pyridin-3-yl)-7-methyl-1,7-dihydro-1,7-naphthyridin-8(2H)-one**, target bromodomain and extra-terminal (BET) domain proteins to increase PGRN expression, directly influencing critical processes, such as neurodevelopment and neuroplasticity [107]. **2-(4-(3-fluoroazetidin-3-yl)-4,5-dihydrofuran-2-yl)-8-(4-fluorophenyl)-1,2,3,4-tetrahydroisoquinoline**, which is orally active, specifically promotes PGRN secretion with minimal cytotoxicity and controlled effects on hERG channels, making it a promising therapeutic candidate [108]. Additionally, **methyl 2-oxo-3-(2-phenylbenzo[d]oxazol-5-yl)propanoate** has demonstrated effectiveness in upregulating PGRN protein levels within human cellular models and has shown potential in correcting PGRN protein deficiency in the brains of GRN+/− mice, effectively reversing lysosomal proteome aberrations, a hallmark of FTD [109]. This compound proved to outperform previously identified agents, like suberoylanilide hydroxamic acid, establishing its potential as a lead for further exploration of PGRN’s cellular functions in FTD and advancing drug development initiatives targeting this condition.

Lastly, conformational allosteric activators have been recognized for their capacity to upregulate PGRN levels. PGRN localizes to lysosomes, associating with prosaposin (PSAP), a protein crucial for glycosphingolipid degradation. Notably, the small molecule AZP2006 binds the PGRN/PSAP complex within lysosomes, stimulating PGRN release and promoting neuronal outgrowth in experimental models. Research has uncovered that AZP2006 interacts with the PGRN and PSAP complex within lysosomal compartments [110]. At nanomolar concentrations, AZP2006 has been shown to stimulate the release of PGRN and promote neurite outgrowth in vitro. PGRN localizes to lysosomes, associating with PSAP, a protein crucial for glycosphingolipid degradation. Notably, AZP2006 exhibits a high affinity for lysosomal vesicles and accumulates within these organelles. Through Fluorescent Microscale Thermophoresis (MST) competition assays, AZP2006 was demonstrated to bind to the PGRN/PSAP complex with greater affinity than to PSAP alone, without measurable interaction with PGRN in isolation. This suggests that the binding of AZP2006 to the PGRN/PSAP complex may be regulated by the conformational state of the complex within the lysosome. AZP2006 has successfully passed good laboratory practice toxicology studies and has undergone human testing (Phases 1 and 2) [111].

**Figure 6:**
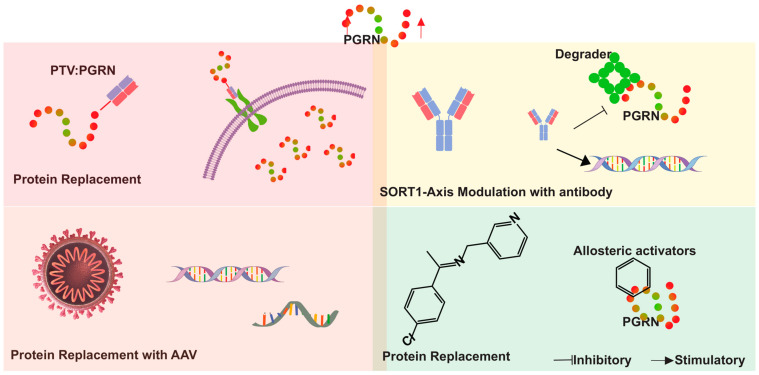
Therapeutic approaches for progranulin (PGRN) augmentation in neuro-degenerative diseases. The figure illustrates four primary strategies. (1) Direct protein replacement therapy using protein transport vehicle (PTV) technology to deliver PGRN across the target organ. (2) Gene therapy approaches utilizing adeno-associated viruses (AAVs) to deliver functional GRN genes and express the PGRN in the target district. (3) Targeting the PGRN-SORT1 axis and the PGRN modulator depicted by antibodies and small molecule inhibitors interfering with PGRN degradation and enhancing the expression of PGRN. (4) Allosteric activators modulate PGRN levels through conformational changes. These strategies collectively aim to increase PGRN levels (indicated by the red arrow), addressing deficiencies associated with conditions such as frontotemporal dementia (FTD).

The majority of therapeutic studies centered on PGRN augmentation—including direct protein replacement (e.g., DNL593), gene therapies (e.g., PR006, AVB-101), and antibody-based SORT1 blockade (e.g., AL001)—have been developed and trialed in the context of neurological disorders. As yet, there are no published clinical trials or human studies evaluating these strategies for cardiovascular indications, highlighting a key translational gap that needs to be addressed in future research. The innovative approaches outlined herein provide a robust foundation for developing effective treatments aimed at mitigating the impact of PGRN-related pathologies (Figure 6).

## 5. Discussion

The multifaceted role of PGRN in CVD has garnered increasing attention as researchers seek novel therapeutic targets to mitigate the impact of this leading cause of morbidity and mortality worldwide. PGRN’s protective mechanisms are primarily attributed to its ability to modulate inflammatory pathways, enhance mitochondrial function, and promote vascular integrity. By interacting with critical signaling pathways, such as PI3K/Akt and NF-κB, PGRN not only mitigates oxidative stress but also fosters an environment conducive to cardiac repair following ischemic injury. This is particularly evident in myocardial infarction (MI), where PGRN levels correlate positively with the severity of coronary artery disease, suggesting its potential as a biomarker for disease severity; conversely, **low baseline PGRN levels may indicate increased susceptibility**.

Moreover, PGRN’s involvement in various cardiac pathologies extends beyond ischemic events to conditions such as sepsis-induced cardiomyopathy and diabetes-induced cardiomyopathy. PGRN exhibits anti-inflammatory and anti-oxidant properties, which are crucial for maintaining cardiac function. The regulation of macrophage polarization and recruitment by PGRN is particularly noteworthy, as these processes are vital for host defense during inflammatory states. Furthermore, PGRN’s role in lipid metabolism and vascular smooth muscle cell behavior underscores its complexity in influencing atherogenesis and vascular homeostasis. The protective effects of PGRN in these conditions highlight its potential as a therapeutic target for preventing adverse cardiac remodeling and improving patient outcomes.

Recent advancements in therapeutic strategies aimed at augmenting PGRN levels have demonstrated considerable promise. Approaches such as direct protein replacement therapy, gene therapy utilizing adeno-associated viruses, and targeting the PGRN–SORT1 axis are being explored to enhance PGRN availability and functionality. These strategies not only aim to restore normal PGRN levels but also to mitigate the pathological consequences associated with its deficiency. The ongoing research into PGRN’s multifaceted roles in cardiovascular health and disease paves the way for innovative therapeutic interventions that could significantly improve the management of CVD, ultimately enhancing patient care and outcomes in this critical area of public health.

## Data Availability

The original contributions presented in this study are included in the article. Further inquiries can be directed to the corresponding author.

## Abbreviations

The following abbreviations are used in this manuscript:

Abbreviation	Full Form
PGRN	Progranulin
CVD	Cardiovascular Disease
MI	Myocardial Infarction
I/R	Ischemia-Reperfusion
PCI	Percutaneous Coronary Intervention
TNF	Tumor Necrosis Factor
TNFR	Tumor Necrosis Factor Receptor
PI3K	Phosphoinositide 3-Kinase
Akt	Protein Kinase B
NF-κB	Nuclear Factor Kappa-Light-Chain-Enhancer of Activated B Cells
VSMC	Vascular Smooth Muscle Cell
eNOS	Endothelial Nitric Oxide Synthase
MCP-1	Monocyte Chemoattractant Protein-1
IL-10	Interleukin-10
HDL	High-Density Lipoprotein
LDL	Low-Density Lipoprotein
agLDL	Aggregated Low-Density Lipoprotein
SIC	Sepsis-Induced Cardiomyopathy
CAVD	Calcific Aortic Valve Disease
hVICs	Human Valve Interstitial Cells
BMP	Bis(mono-acyl-glycero)phosphate
GCase	Glucocerebrosidase
TFEB	Transcription Factor EB
LAMP-1	Lysosomal-Associated Membrane Protein 1
OCR	Oxygen Consumption Rate
ROS	Reactive Oxygen Species
AAV	Adeno-Associated Virus
FTD	Frontotemporal Dementia
CSF	Cerebrospinal Fluid
SORT1	Sortilin
PSAP	Prosaposin
BET	Bromodomain and Extra-terminal Domain
hERG	Human Ether-à-go-go-Related Gene

## Appendix A

**Table A1:** Summary of PGRN-related therapeutic strategies under investigation.

Strategy Type	Therapeutic Candidate	Mechanism of Action	Clinical Development
Direct Protein Replacement	AZP2006	Binds to the PGRN/PSAP complex in lysosomes, promoting PGRN release and neurite outgrowth.	Completed Phase 1-2 trials.
Allosteric Activators	AZP2006	Promotes PGRN release by binding to the PGRN/PSAP complex within lysosomal compartments.	Completed Phase 1-2 trials.
Gene Therapy	AAV-delivered GRN	Delivers functional GRN genes or promotes PGRN secretion through compounds like **5** and **6**.	Preclinical and early clinical stages.
Targeting SORT1 Axis	AL001	Monoclonal antibody that blocks the interaction between SORT1 and PGRN, increasing extracellular PGRN levels.	Increased PGRN in plasma/CSF; studies in mutation carriers.
Inhibitors of SORT1-PGRN Interaction	Compounds **1**–**3**	Small molecule inhibitors disrupting the SORT1-PGRN complex to increase extracellular PGRN.	Compound 3 shows low IC50, preclinical stage.
Modulators of Expression/Secretion	Compounds **4**–**6**	Modulate pathways like BET proteins or directly enhance PGRN secretion.	Preclinical; some in early clinical trials.
